# Whole mitochondrial genome analysis of the Daur ethnic minority from Hulunbuir in the Inner Mongolia Autonomous Region of China

**DOI:** 10.1186/s12862-022-02019-4

**Published:** 2022-05-18

**Authors:** Chi-Zao Wang, Xue-Er Yu, Mei-Sen Shi, Hui Li, Shu-Hua Ma

**Affiliations:** 1 Department of Radiology, The First Affiliated Hospital of Shantou University Medical College, Shantou, 515041 China; 2 Shantou University Medical College, Shantou, 515041 Guangdong China; 3 Laboratory of Medical Molecular Imaging, The First Affiliated Hospital of Shantou University Medical College, No. 57 Changping Road, Shantou, 515041 Guangdong China; 4 MOE Key Laboratory of Contemporary Anthropology, School of Life Sciences, Fudan University, 200438 Shanghai, China; 5 Criminal Justice College of China University of Political Science and Law, Beijing, 100088 People’s Republic of China; 6 Shanxi Academy of Advanced Research and Innovation, Fudan-Datong Institute of Chinese Origin, Datong, 037006 China

**Keywords:** Daur, Mongolic-speaking groups, Whole mtDNA sequence, Heilongjiang River basin, Northern East Asia

## Abstract

**Background:**

Mitochondrial DNA (mtDNA) variations are often associated with bioenergetics, disease, and speciation and can be used to track the history of women. Although advances in massively parallel sequencing (MPS) technology have greatly promoted our understanding of the population’s history (especially genome-wide data and whole Y chromosome sequencing), the whole mtDNA sequence of many important groups has not been fully studied. In this study, we employed whole mitogenomes of 209 healthy and unrelated individuals from the Daur group, a Mongolic-speaking representative population of the indigenous groups in the Heilongjiang River basin (also known as the Amur River basin).

**Results:**

The dataset presented 127 distinct mtDNA haplotypes, resulting in a haplotype diversity of 0.9933. Most of haplotypes were assigned to eastern Eurasian-specific lineages, such as D4 (19.62%), B4 (9.09%), D5 (7.66%) and M7 (4.78%). Population comparisons showed that the Daurians do have certain connections with the ancient populations in the Heilongjiang River basin but the matrilineal genetic composition of the Daur group was also greatly influenced by other non-Mongolic groups from neighboring areas.

**Conclusions:**

Collectively, the whole mtDNA data generated in the present study will augment the existing mtDNA database. Our study provides genetic links between the Daur population and the aborigine peoples from Siberia and the Amur-Ussuri Region. But on the whole, compared with other Mongolic-speaking groups, the modern Daur population is closer to the East Asian ancestry group.

**Supplementary information:**

The online version contains supplementary material available at 10.1186/s12862-022-02019-4.

## Background

The Daur minority is one of the important members of the Mongolic-speaking population. They originally lived on the north beach of the Heilongjiang River (Amur River) [1]. After the 17th century, they gradually moved to Hulunbuir, Qiqihar and other settlements on the south beach of the Heilongjiang River [2]. A small number of them even migrated to Tacheng Prefecture in Xinjiang Province with their families as government troops of the Qing Dynasty[3]. The Daur ethnic group has long intermingled with the Ewenk and Oroqen ethnic groups, two other officially recognized ethnic groups in China, who are members of the Tungusic-speaking ethnic populations. They were known as the Suolun in the Qing Dynasty and are now called the Three Minorities in Inner Mongolia [3].

The Daur ethnic group has appeared in many important genetic studies as one of the representatives of Mongolic-speaking populations and indigenous groups from the Heilongjiang River basin [4–6]. Our previous studies elaborated on the paternal phylogenetic relationship between the Daur group, other Mongolic-speaking populations and Tungusic-speaking populations (including the Aisin Gioro family) by analyzing their Y-STR genetic polymorphisms and whole Y-chromosome sequences [7–11]. Recent genome-wide studies have further revealed the high level of genetic continuity of indigenous populations from the Heilongjiang River basin (including the Daur ethnic group) over at least the last 14,000 years and their distinct phylogenetic position in the genetic structure of human populations in East Asia [6, 12, 13]. However, previous studies on the Daur group from the perspective of maternal inheritance were relatively limited in terms of sample size and merely based on partial sequence polymorphisms, such as hypervariable segments I and II (HVS-I and HVS-II, respectively) and the control region (CR) [14–16]. Two early genetic studies established a certain genetic relationship between the modern Daur group and the ancient Khitan, which is one of the most significant findings of ethnic studies in China [15, 16]. Therefore, expanding the sample size and introducing whole mitochondrial genome analyses will undoubtedly contribute to a more comprehensive understanding of the maternal genetic background of the Daurians.

In this study, the whole mitochondrial genomes of 209 healthy and unrelated Daur individuals from Northeast China were sequenced by massive parallel sequencing (MPS) on the HiSeq X Ten system (Illumina, San Diego, CA, USA). Based on the sequencing data, we analyzed the haplogroup distribution and genetic diversity of the maternal genetic structure of the Daur group. To shed more light on the genetic relationship of the Daur group with worldwide populations, especially other neighboring/linguistically close populations and some related ancient groups, we conducted comprehensive population genetic analyses via Principal Component Analysis (PCA) [17]and other methods.

## Methods

### Samples, DNA extraction and quantification

A cohort of 209 unrelated Daur individuals (84 females and 125 males) was collected after receiving informed consent. The individuals were considered autochthonous if their ancestors had lived in Hulunbuir, Inner Mongolia Autonomous Region of China, for at least three generations. Written informed consent was obtained from all participants, and the ethics committee of School of Life Sciences, Fudan University, Shanghai, People’s Republic of China approved this study.

Genomic DNA was extracted from blood samples using a DP-318 Kit (Tiangen Biotechnology, Beijing, China) according to the manufacturer’s protocol. The quantity of gDNA was measured with a NanoDrop ND-1000 (NanoDrop Technologies, Wilmington, DE, USA) according to the manufacturer’s protocol. In consideration of the requirements of downstream processing, the gDNA was normalized to 0.1 ng/µL and stored at − 20 °C until amplification.

### Library construction and workflows for next-generation sequencing

DNA libraries were constructed using an MtDNA Library Preparation Kit 2.0 (Enlighten Biotech, Shanghai, China) and a WhoChrMT kit (Enlighten Biotech, Shanghai, China). PCR amplification was performed in a final volume of 30 µL containing 10 ng of template DNA, 5 µL RealCapChrMT Mix and 10 µL 3×EnzymeHF. Total reaction volumes were adjusted with nuclease-free water. The PCR was performed under the following conditions: enzyme activation for 3 min at 98 °C, 13 cycles of 20 s at 98 °C and 4 min at 58 °C, 7 cycles of 20 s at 98 °C and 1 min at 72 °C, 2 min at 72 °C followed by a 10 °C hold. The PCR products were purified with Agencourt AMPure XP beads (Beckman Coulter). Then, a second round of PCR amplification was carried out to introduce adapters and barcodes. The reaction volume (30 µL) was comprised of 10 µL 3×EnzymeHF, 18 µL nuclease-free water, 1 µL primer mix and 1 µL barcode mix. The PCR was performed under the following conditions: enzyme activation for 2 min at 98 °C, 7 cycles of 15 s at 98 °C, 15 s at 58 °C and 30 s min at 72 °C, extension for 2 min at 72 °C followed by a 10 °C hold. After purification, the libraries were pooled to a final concentration of 20 pM. Sequencing was performed on the Illumina HiSeq X Ten platform (Illumina, San Diego, CA, USA) with the corresponding Reagent Kit (PE150).

### Sequencing data analysis

The sequence data obtained from the Illumina HiSeq X Ten platform (Illumina, San Diego, CA, USA) were automatically analyzed by base recognition and converted into the original sequences in FASTQ format. First, redundant primers and indexes in the initial offline data were removed by cutadapt software [18]. Second, low-quality reads were filtered by Trimmomatic software [19]. To ensure the successful alignment of the loop amplification captured sequence, the final cleaned files were mapped to the revised Cambridge Reference Sequence [20] plus 64 bp (rCRS + 64 bp) using the Burrows-Wheeler Aligner [21] to generate the binary alignment/map (BAM) file. The sequences were also compared with the human reference genome hg19 to filter nuclear copies of mtDNA (NUMTs) [22]. We used Bedtools [23] to extract all reads that were successfully mapped to the HG19 reference genome from the BAM files in the previous step and then realigned them to rCRS + 64 bp to generate new BAM files using Bowtie2 software [24]. Then, SAMtools [25] and VarScan [26] were used to identify the mutation sites and output variants in VCF format files. Finally, BCFTools [25] was used to generate the consensus sequence (FASTA).

### Haplogroup assignment and genetic diversity analysis

Sequencing performance was evaluated by read depth. The mtDNA haplogroups were determined using HaploGrep 2 [27] based on PhyloTree build 17 [28] and reconfirmed using the updated query engine (SAM2) built into EMPOP [29]. With reference to PhyloTree build 17, we constructed a simplified phylogenetic tree that showed the distribution of the coarse haplogroups. Haplotype diversities were calculated according to Nei’s formula [30]. The discrimination capacity (DC) was also calculated as an important diversity parameter [31]. To show the differences in the genetic diversity of the different mitochondrial regions, haplotype-based analyses were repeated for the control region (CR, 16,024 to 576) and hypervariable segment I (HVS1, 16,024 to 16,488).

### Population comparisons

To investigate the genetic relationship between the Daur group and other populations around the world, the whole mitochondrial genomes dataset was collected from 128 worldwide populations (Additional file 1: Table S1). In particular, we required the group size to be greater than 15 to avoid artificially low genetic diversity. Subsequently, the genetic background of the Daur group was analyzed by typical Principal Component Analysis (PCA) with the R statistical package (https://www.r-project.org/) based on haplogroup frequencies (Additional file 1: Table S2). *AncestryPainter* was used to illustrate the haplogroup sharing and ancestry composition of populations with a rounded and nice-looking graph [32]. For some mtDNA haplogroups of particular interest, the network analysis was constructed using the median-joining method in the Popart software [33, 34]. We also compared all Daur mitogenomes and published raw sequences (both ancient and modern, Additional file 1: Tables S1, S3) in the hope of identifying some perfect matches.

## Results and discussion

### Sequence performance

The average mapped reads were 139,681 per sample, and the overall mean read depth was 1260X ± 422X (mean ± SD) per individual. The variants recommended by EMPOP as well as the haplogroup information and the mean sequencing depth of 209 Daur individuals are presented in Additional file 1: Table S4.

### Haplogroup distribution

**Fig. 1:**
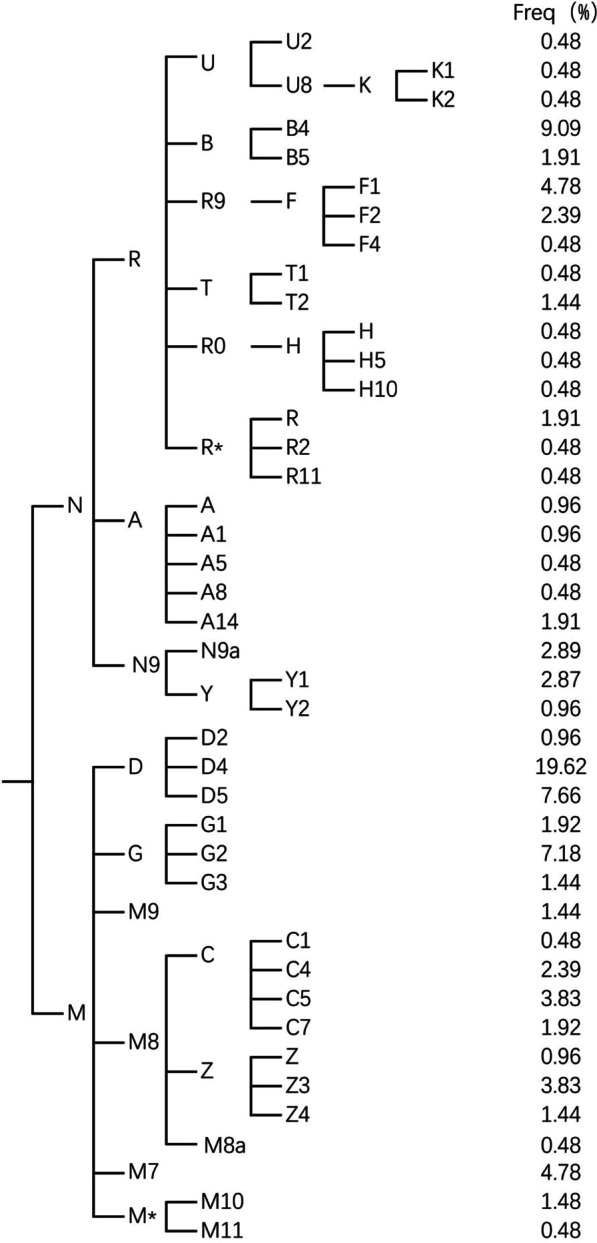
The phylogenetic relationship of coarse mtDNA haplogroups in this study and their clan-based frequencies among the Daur group

Figure 1 presents a simplified phylogenetic tree that shows the distribution of the coarse haplogroups, and the detailed typing results are shown in Additional file 1: Table S4. In general, the matrilineal component of the Daur group was predominantly comprised of the eastern Eurasian-specific component (89.21%), represented by haplogroups D (28.24%), G (10.54%), B (10%), C (8.62%), R9 (7.65%), N9 (6.92%), Z (6.23%), A (4.79%), M7 (4.78%) and M9 (1.44%) [35, 36]. The remaining samples consisted of haplogroups U (1.44%), T (1.92%) and H (1.44%), which are generally confined to the European region [36, 37], and a few root types (R* and M*). Among these haplogroups, C and D have distinct Asian characteristics, and more than half of the northern Asian pool of human mtDNA is fragmented into their subclades [35, 38]. In the Daur population we studied, haplogroup C consisted of four sister subclades, C1 (0.48%), C4 (2.39%), C5 (3.83%) and C7 (1.92%), while haplogroup D consisted of three sister subclades, D2 (0.96%), D4 (19.62%) and D6 (7.66%). Notably, haplogroup D4 not only has a high frequency but also contains a total of 28 abundant downstream clades (Additional file 1: Table S4). Some subbranches of haplogroup D4 have very distinctive geographical distributions and are of great significance for the study of the demographic history of Asia [38, 39]. For example, haplogroup D4j (2.87% in this study) demonstrated a more southern geographic distribution, and haplogroup D4e4a (0.48% in this study) was mostly found in the Subarctic and Arctic regions [40]. According to previous studies, haplogroups B (10% in this study) and G (10.54% in this study) are also frequent in Mongolic-speaking groups [35, 41].

On the whole, the Daur population in this study embodies distinct regional and ethnic characteristics. Compared with earlier studies on Daur mtDNA [14–16], our research showed some changes in some haplogroup frequency distributions and detected some types that were not previously found in the Daur population (U, F, H, etc.), which could be attributed to the larger sample size and more advanced full mtDNA sequencing methods used in this study.

### Genetic diversity analysis

Based on whole mtDNA sequence data, a total of 127 different haplotypes were identified from the 209 unrelated Daur samples, of which 81 (63.78%) were unique. Although close matrilineal relatives (first to three degrees) were excluded, 61.24% of the total samples still shared haplotypes with others. It is worth noting that the haplotypes belonging to M7b1a1+(16,192), G2a1 and Z3d were shared by 6 individuals. Moreover, one haplogroup was shared between five individuals, seven were shared between four individuals, seven were shared between three individuals and 28 were shared between two individuals. The overall haplogroup diversity was calculated as 0.9933 with a discrimination capacity of 60.77%. Additional file 1: Table S5 summarizes the above results. Repeated analysis based on CR and HVS1 showed that whole mtDNA sequence data decreased the number of shared haplotypes and increased the number of unique haplotypes. This is reflected in the discriminatory capacity increasing from 53.11% with the HVS1 haplotypes and 54.55% with the CR haplotypes to 60.77% with the whole mtDNA sequence for the Daur samples (Additional file 1: Table S5). These results indicate that the whole mtDNA sequence data offer a high power of discrimination and can be useful for genetic investigation and maternal lineage research in the Daur minority.

Of course, the genetic diversity of maternal genetic markers was slightly lower than that of paternal genetic markers, which is more due to the limitations of mitochondrial genetic markers themselves. In our previous study of genetic polymorphisms of 27 Yfiler^®^ Plus loci in the Daur group, a total of 196 different haplotypes were observed in the sample of 203 Daur individuals, and the overall haplotype diversity was calculated as 0.9997 with a discrimination capacity of 0.9655 [7]. Our other two studies based on Y-STR/Y-SNP and Y-chromosome sequencing provided rich details on the paternal genetic diversity of the Daur group [8–10].

### Population comparisons

#### PCA

**Fig. 2:**
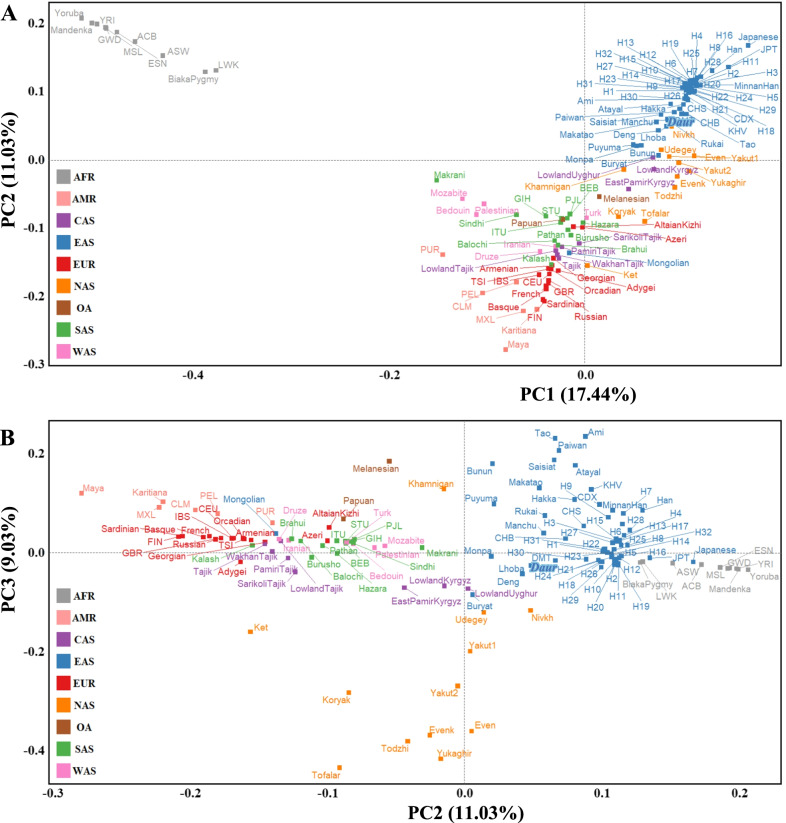
Principal component (PC) analyses

In our PCA results, 37.5% of the genetic variations were extracted by the first three components (Fig. 2). The African ancestry (AFR) populations can be separated clearly by PC1 and PC2, while the other large groups are closely related and even overlap. Our Daur population was clustered with other populations of the East Asian ancestry(EAS). As for the other two Mongolic-speaking populations, the Buryat population was located at the boundary between the groups of the East Asian ancestry (EAS) and the North Asia Ancestry(NAS), while the Mongolian population was closely related to groups of the West Asian Ancestry (WAS) and the Central Asian Ancestry (CAS). The distribution positions of the three Mongolic-speaking populations in the PCA map was roughly consistent with their geographic areas, which may reflect the maternal genetic contributions from different groups during the migration and development of the Mongolic-speaking groups.

#### Haplogroup sharing analysis

**Fig. 3:**
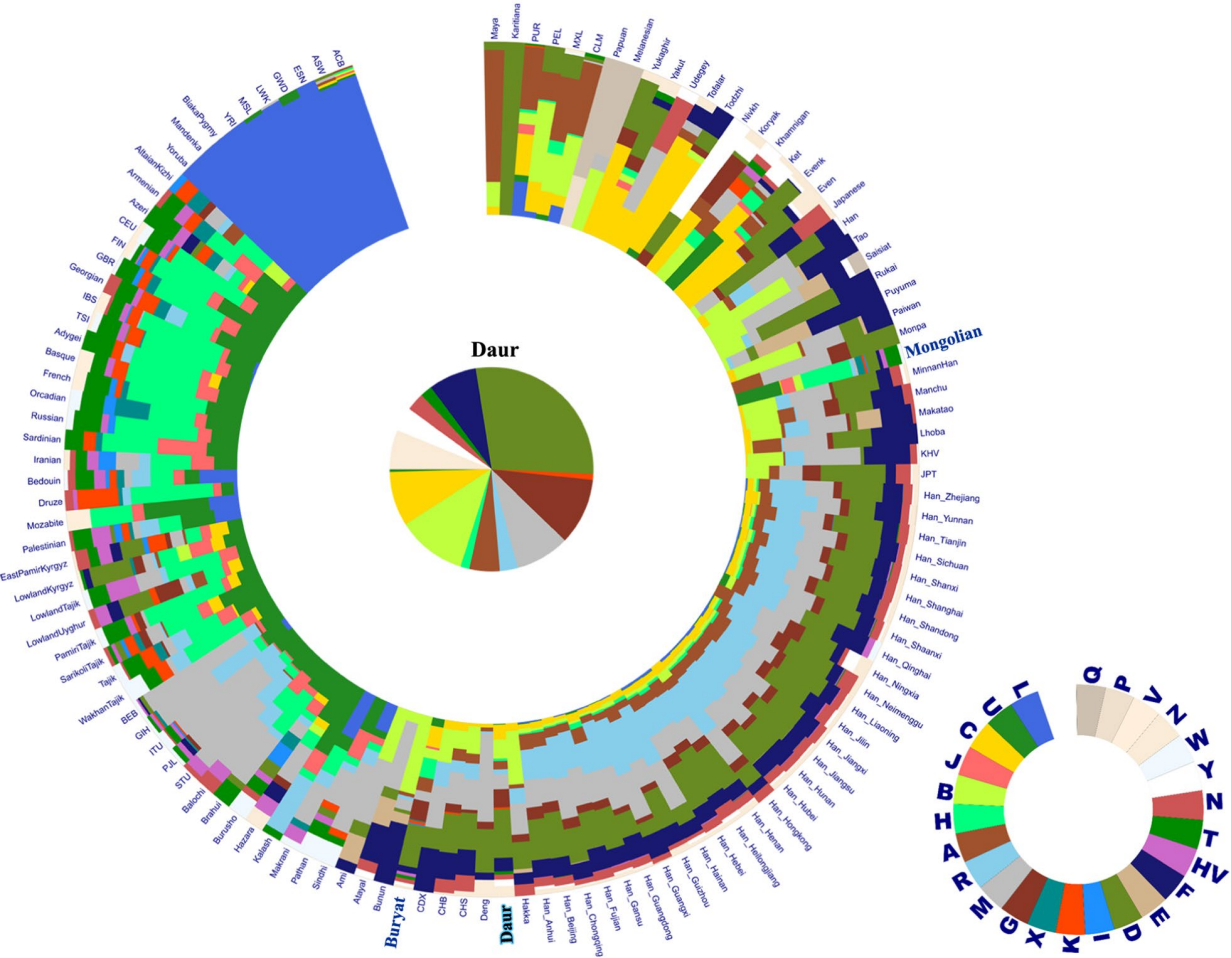
Haplogroup sharing analysis

In the data visualization of the haplogroup sharing analysis (Fig. 3), the Daur population also showed a similar population structure to the EAS groups represented by a series of Han ethnic populations, but the proportion of haplogroup B and R was quite different from that of the Han groups. In the comparison with the other two Mongolic-speaking populations, the haplogroup H ratio of the Daur population (1.44%) is significantly lower than that of the Buryat population (11.52%) and the Mongolian population (28.57%). Haplogroup H could be regarded as one of the representative haplogroups of European ancestral (EUR) groups, often accounting for 40% or more of the total haplogroups. Therefore, similar to the PCA reasults, the Daur population was closer to the eastern Eurasia groups, while the other two Mongolic-speaking populations were closer to the western Eurasia groups.

#### Network analysis

**Fig. 4:**
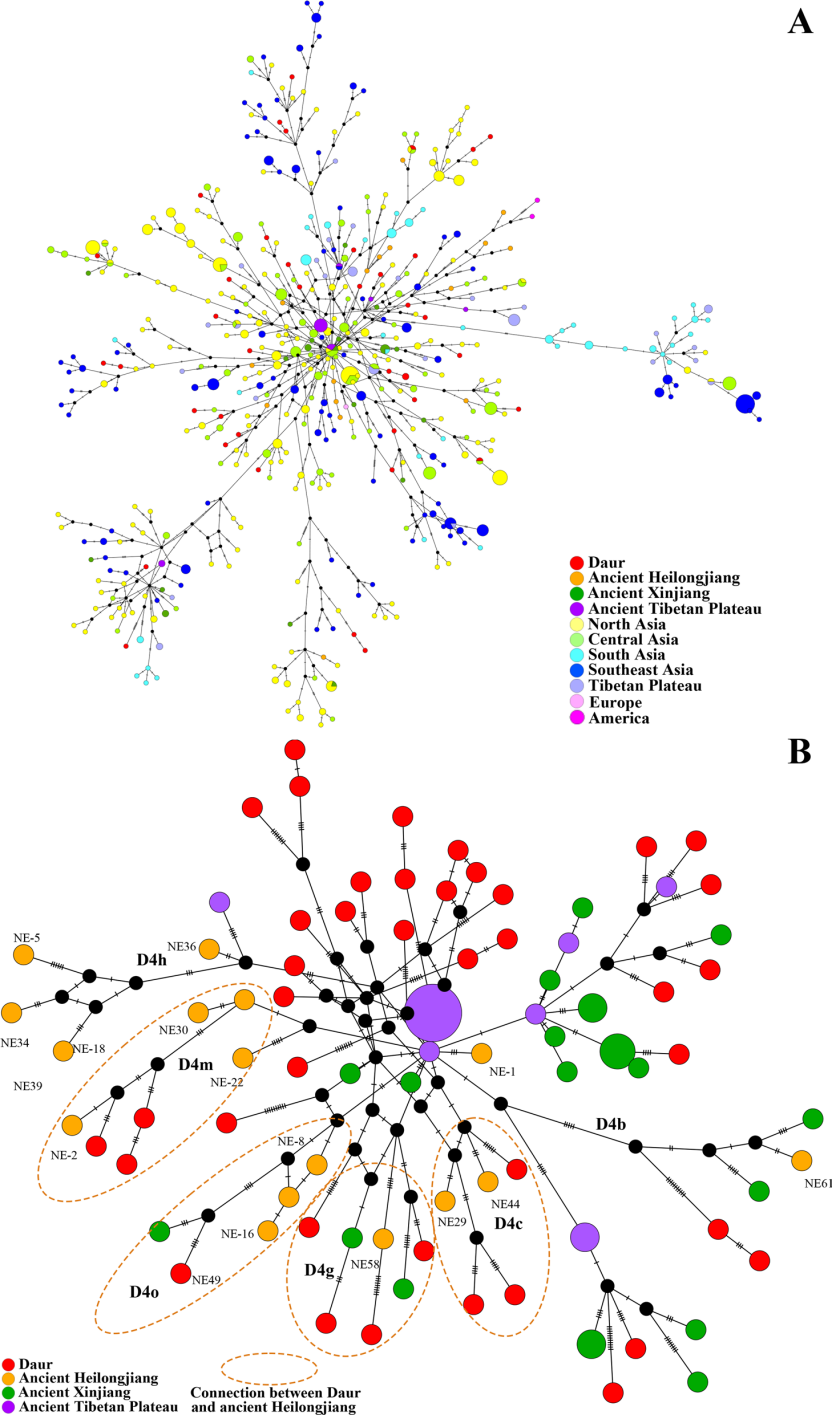
Median-joining haplogroup D4 networks. **A** Ancient and modern samples. **B** The modern Daur and ancient samples. Note 1: According to the naming method of the original author, samples from the ancient Heilongjiang River basin have been specially labeled [13]. The larger the number, the older the age. Note 2: Black internal nodes only represent data structure bifurcation points, not actual samples

As mentioned above, haplogroup D4 not only has a high frequency (19.62%) but also contains abundant downstream clades in the Daur samples. According to previous studies based on partial sequences, D4 is also the high-frequency type of several ancient ethnic groups in Northeast China [42–44]. In the latest genome-wide study of northern East Asia, D4 also accounted for the majority of the detected samples in the ancient Heilongjiang River basin(66.67%, 16/24) [13]. We collected relevant available full sequence data (Additional file 1: Table S3) and constructed networks (Fig. 4). In Fig. 4A, the Daur samples came from scattered sources, showing connections with multiple regions of Asia. When we focused on the genetic connection between the Daur samples and ancient samples, we found that most samples from the ancient Heilongjiang River basin had close connections with samples of Daur (Fig. 4A, B), and concentrated in haplogroups D4m, D4o, D4g and D4c. Haplogroup D4h, another high-frequency type in ancient Heilongjiang River basin populations, has not been detected in the modern Daur group which also makes sense that D4h is a distinctive native American type that may not have been involved in the late demographic history of northern East Asia [45]. In other words, the network analysis shows that the Daurians do have certain connections with the ancient populations in the Heilong River basin, but in the development process of the Daurians, they also absorbed a large number of female population from other sources. As to whether the modern Daur group has the closest matrilineal genetic connection with the ancient Heilongjiang population, we will collect more complete mitochondrial sequence data and carry out it in detail in follow-up studies.

#### Perfectly matched sequences

After comparing all raw whole sequences (both ancient and modern), we found two perfect matches between the Daur mitogenomes and published Buryat sequences (Table 1), DMT040-Buryat 643 [46] in haplogroup A5c and DMT185-Buryat 618 [46] in haplogroup A8a1, respectively. Haplogroup A5c includes one raw sequence from the Khamnigan population [46], while haplogroup A8a1 includes another perfect match (ald1-sun30) formed in the Yakut population [47]. The discovery of these perfectly matched sequences reflects the genetic connection between the Daur population and other aborigine peoples from Siberia and the Amur-Ussuri Region. Although haplogroup A5c and A8a1 are not the dominant types in the present Daur population, they may be genetic traces from early ancestors and exist in low-frequency form. Of course, more perfectly matched sequences may be found in the future with the increasing abundance of whole mitochondrial genome data.

**Table 1:** Summary of perfected matched sequences

Haplogroup	A5c	A8a1
Perfect match	DMT040 and Buryat 643	DMT185 and Buryat 618
		ald1 and sun30
The other raw sequnces	Khamnigan 43	-

## Conclusions

The present study provided the first set of whole mitochondrial genome data of 209 Daur individuals residing in Northeast China. The investigation of the Daur maternal lineages revealed that the vast majority of haplogroups belong to the eastern Eurasian-specific component. Population analyses showed that the Daurians do have certain connections with the ancient populations in the Heilongjiang River basin but the matrilineal genetic composition of the Daur group was also greatly influenced by other non-Mongolic groups from neighboring areas. This study also shows that whole mitochondrial sequence data can improve the resolution and offers a high power of discrimination in maternal studies by comparison of whole and partial sequence data in genetic diversity and population comparative analyses. Overall, the mitogenomes generated in the present study will augment the existing Daur mtDNA database, which provides a deeper understanding of the genetic composition of the Daur group and could potentially be useful for regional-specific and prerequisite references for forensic, genealogical, and evolutionary purposes.

## Supplementary Information

**Additional file 1: Table S1**. Reference groups used in PCA and haplogroup sharing analysis. **Table S2**. Coarse haplogroup frequencies. **Table S3**. References used in networks. **Table S4**. The detail information for the full mtDNA sequences observed in 209 Daur individuals. **Table S5**. Diversity indices for the Daur population obtained with different mtDNA regions.

## Data Availability

The 209 novel Daur complete mtDNA sequences have been uploaded to the Genome Sequence Archive (GSA) in the BIG Data Center (Members BIGDC 2017), Beijing Institute of Genomics (BIG), Chinese Academy of Sciences (http://bigd.big.ac.cn/gsa-human) .The assigned accession of the submission is: HRA001624.

## Abbreviations

MtDNA: Mitochondrial DNA
MPS: Massively parallel sequencing
HVS-I and HVS-II: Hypervariable segments I and II
CR: Control region
PCA: Principal component analysis
DC: Discrimination capacity
N-J: Neighbor-joining
